# Genetic Variations in *EIF2AK3* are Associated with Neurocognitive Impairment in People Living with HIV

**DOI:** 10.1007/s11481-024-10125-x

**Published:** 2024-05-25

**Authors:** Cagla Akay-Espinoza, Sarah E.B. Newton, Beth A. Dombroski, Asha Kallianpur, Ajay Bharti, Donald R. Franklin, Gerard D. Schellenberg, Robert K. Heaton, Igor Grant, Ronald J. Ellis, Scott L. Letendre, Kelly L. Jordan-Sciutto

**Affiliations:** 1 Department of Oral Medicine, School of Dental Medicine, University of Pennsylvania, 240 S. 40th St, Rm 312 Levy, Philadelphia, PA 19104 USA; 2 Department of Pathology and Laboratory Medicine, The Perelman School of Medicine, University of Pennsylvania, Philadelphia, PA USA; 3 Genomic Medicine Institute, Lerner Research Institute, Cleveland Clinic, Cleveland, OH USA; 4 Department of Molecular Medicine, Cleveland Clinic Lerner College of Medicine, Case Western Reserve University, Cleveland, OH USA; 5 Departments of Medicine, University of California, San Diego, CA USA; 6 Department of Psychiatry, University of California, San Diego, CA USA; 7 Department of Neurosciences, University of California, San Diego, CA USA

**Keywords:** *EIF2AK3*, Haplotype, HIV, Integrated stress response, Neurocognitive impairment, Single nucleotide variant

## Abstract

Based on emerging evidence on the role for specific single-nucleotide variants (SNVs) in *EIF2AK3* encoding the integrated stress response kinase PERK, in neurodegeneration, we assessed the association of *EIF2AK3* SNVs with neurocognitive performance in people with HIV (PWH) using a candidate gene approach. This retrospective study included the CHARTER cohort participants, excluding those with severe neuropsychiatric comorbidities. Genome-wide data previously obtained for 1047 participants and targeted sequencing of 992 participants with available genomic DNA were utilized to interrogate the association of three noncoding and three coding *EIF2AK3* SNVs with the continuous global deficit score (GDS) and global neurocognitive impairment (NCI; GDS ≥ 0.5) using univariable and multivariable methods, with demographic, disease-associated, and treatment characteristics as covariates. The cohort characteristics were as follows: median age, 43.1 years; females, 22.8%; European ancestry, 41%; median CD4 + T cell counts, 175/µL (nadir) and 428/µL (current). At first assessment, 70.5% used ART and 68.3% of these had plasma HIV RNA levels ≤ 200 copies/mL. All three noncoding *EIF2AK3* SNVs were associated with GDS and NCI (all *p* < 0.05). Additionally, 30.9%, 30.9%, and 41.2% of participants had at least one risk allele for the coding SNVs rs1805165 (G), rs867529 (G), and rs13045 (A), respectively. Homozygosity for all three coding SNVs was associated with significantly worse GDS (*p* < 0.001) and more NCI (*p* < 0.001). By multivariable analysis, the rs13045 A risk allele, current ART use, and Beck Depression Inventory-II value > 13 were independently associated with GDS and NCI (*p* < 0.001) whereas the other two coding SNVs did not significantly correlate with GDS or NCI after including rs13045 in the model. The coding *EIF2AK3* SNVs were associated with worse performance in executive functioning, motor functioning, learning, and verbal fluency. Coding and non-coding SNVs of *EIF2AK3* were associated with global NC and domain-specific performance. The effects were small-to-medium in size but present in multivariable analyses, raising the possibility of specific SNVs in *EIF2AK3* as an important component of genetic vulnerability to neurocognitive complications in PWH.

**Graphical Abstract:**

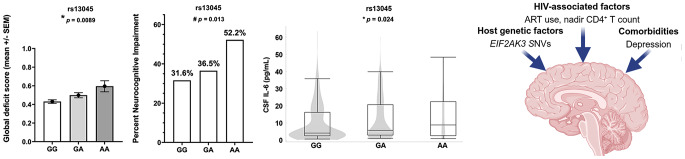

**Supplementary Information:**

The online version contains supplementary material available at 10.1007/s11481-024-10125-x.

## Introduction

Despite vastly improved health outcomes with antiretroviral therapy (ART), up to 50% of people with human immunodeficiency virus (HIV) infection (PWH) continue to experience neurocognitive (NC) impairment (NCI) and other neurological disorders (Heaton et al. 2015; Wei et al. 2020). Milder forms of NCI persist and may worsen even when HIV RNA is suppressed by ART (Heaton et al. 2015). Progression to more disabling NC disorders is more likely in PWH with mild, even asymptomatic, NCI than in unimpaired PWH (Grant et al. 2014). Accumulating evidence also supports that neuropsychiatric disorders such as depression and aging-related disorders are more common in PWH (Cook et al. 2018; Lu et al. 2019; Valcour et al. 2011).

Suppressive ART is currently the only proven intervention to prevent or improve NCI in PWH. Several factors increase the risk for NCI, including lower nadir CD4^+^ T cell count, metabolic syndrome, hepatitis C virus (HCV) coinfection, depression, and older age (Hua et al. 2013; Martin-Thormeyer and Paul 2009). To date, clinical trials have not identified interventions that further improve on the benefits of ART. Mechanistic biomarkers of NC vulnerability should enable earlier diagnosis of NCI and new therapeutic interventions.

While some of the risk factors associated with NCI, such as sex and genetic factors, may be known at the time of diagnosis and can be considered time-invariable, others, such as metabolic syndrome, arise later and can vary over time. While knowledge of risk factors is paramount for the successful prevention and management of NCI, identification of host factors that are predictive for these at the time of, or soon after, HIV diagnosis, could have an impact by providing opportunities to implement adjunctive therapies. Conversely, genetic vulnerability is well studied in several neurodegenerative disorders, but less is known about the potential contribution of genetic risk factors to HIV-associated NCI (Andres et al. 2011; Kallianpur and Levine 2014; Levine et al. 2009, 2017).

Several of the risk factors for NCI, including HIV replication, older age, and persistent inflammation, induce the integrated stress response (ISR), a ubiquitous cellular response pathway used by cells in the brain and elsewhere to resolve cellular stress and reach homeostasis (Pakos-Zebrucka et al. 2016). The ISR involves the activation of at least one of four sensor kinases, GCN2, HRI, PKR, and PKR-like endoplasmic reticulum kinase (PERK), in response to amino acid or heme deprivation, viral infection, and endoplasmic reticulum (ER) stress, with subsequent activation via multimerization and autophosphorylation. When active, the common target of all four ISR kinases is eukaryotic initiation factor 2α (eIF2α) which, when phosphorylated, attenuates global protein synthesis while upregulating the translation of a subset of transcripts involved in establishing protein homeostasis, such as ATF4 and genes involved in the antioxidant response (Cullinan and Diehl 2004). When ISR activation is prolonged, genes involved in cell death including caspase 4 and CCAAT/enhancer-binding proteins (C/EBP) homologous protein (CHOP) can be induced (Pakos‐Zebrucka et al., 2016); thus, transient ISR activation may be protective but chronic activation may result in cell damage or death (Pakos‐Zebrucka et al., 2016; Verfaillie et al. 2013; Zhang and Kaufman 2008). Importantly, we and others have shown ISR activation, including the increased expression of phospho-PERK and phospho-eIF2 α (peIF2α), to be present in PWH with NCI as well as in people without HIV who have Alzheimer’s disease (AD), Huntington Disease, or progressive supranuclear palsy (PSP) (Akay et al. 2012; Atkin et al. 2008; Gannon et al. 2017; Hoozemans et al. 2009).

Intriguingly, recent studies by Schellenberg and colleagues raise the possibility that variants of *EIF2AK3*, which encodes PERK, may also be a genetic risk factor in neurodegenerative diseases based on the finding that a specific protein-coding haplotype of *EIF2AK3* was associated with increased risk for PSP through genome-wide association and postmortem studies (Hoglinger et al. 2011; Stutzbach et al. 2013). To date, three major coding haplotypes of *EIF2AK3* have been identified; of these, the common haplotypes are A (64%) and B (29%) based on 26 genome-wide association studies (GWASs) (http://www.ncbi.nlm.nih.gov/SNV/), whereas the less common D haplotype has a frequency of 6% in the 1000 Genome data (Table 1). Schellenberg et al. showed that *EIF2AK3* haplotype B harboring minor alleles of three nonsynonymous single nucleotide variants (SNVs), including rs7571971, in the coding region was associated with increased PSP risk (Hoglinger et al. 2011; Stutzbach et al. 2013). The authors also reported that the rs7571971 risk allele was in strong linkage disequilibrium with the haplotype B-coding SNVs. Another study demonstrated that lymphoblastoid cell lines derived from *EIF2AK3* haplotype B-expressing individuals had enhanced PERK activity compared to those lines derived from *EIF2AK3* haplotype A-expressing individuals (Liu et al. 2012); furthermore, PERK inhibition by small-molecule inhibitors or by modulation of eIF2α phosphorylation was reported to alleviate neurological deficits and pathology in small animal models of several neurodegenerative diseases (Devi and Ohno 2014; Mercado et al. 2018; Vaccaro et al. 2013).

**Table 1:** Major haplotypes of *EIF2AK3*

	rs867529-rs13045-rs1805165	Amino acids
Haplotype A	CGT	Ser136-Arg166-Ser704
Haplotype B	GAG	Cys136-Gln166-Ala704
Haplotype D	CAT	Ser136-Gln166-Ser704

In light of the evidence showing increased or sustained PERK activity as detrimental to several critical cellular pathways in a wide range of conditions and based on the possibility of a link between specific minor alleles of *EIF2AK3* and neurodegenerative diseases with features shared by NCI in PWH, we hypothesized that minor *EIF2AK3* alleles such as haplotype B-associated SNVs are a risk factor for NCI in PWH. The objective of the present study was to determine whether minor alleles of *EIF2AK3* SNVs, which were previously reported to be associated with neurodegeneration, correlated with worse NC performance in PWH.

## Materials and Methods

### Study Cohort

This was a retrospective study of data prospectively collected from 1047 PWH who were assessed in the CNS HIV Anti-Retroviral Therapy Effects Research (CHARTER) cohort (Heaton et al. 2015) between 2003 and 2015 and who had genomic DNA available for analysis. Participants who had severe neuropsychiatric comorbidities (e.g., untreated schizophrenia or seizure disorder) were excluded. All participants were comprehensively evaluated with neuromedical and NC assessments at least once, and 566 participants were assessed at least twice (median 7 assessments, interquartile range 4–11). Blood was collected by venipuncture and cerebrospinal fluid (CSF) was collected by lumbar puncture. All study procedures, including genetic testing, were approved by an Institutional Review Board. All participants provided written informed consent for the study procedures, including future use of data, biospecimens, and genetic data.

Genomic data were analyzed in two phases. The first phase included the genome-wide data of 1047 participants in the CHARTER cohort. The data were queried for three noncoding SNVs in *EIF2AK3* (rs6739095, rs1913671, rs11684404), which were previously reported to be in linkage disequilibrium with the three coding SNVs in *EIF2AK3*. In parallel, targeted sequencing (TS) was performed in 992 of the 1047 individuals with available genomic DNA to determine the genotypes of three coding SNVs in *EIF2AK3* (rs867529, rs1805165, and rs13045), which determine the A, B, and D haplotypes of *EIF2AK3*.

### Targeted Sequencing

Genomic DNA samples of the 992 individuals were analyzed using TaqMan SNV genotyping assays (Life Technologies) for rs867529, rs1805165, and rs13045. The assays were performed by polymerase chain reaction according to the previously published conditions (Stutzbach et al. 2013). Genotypes were visualized and called using a 7900HT Fast Real Time PCR system and the allelic discrimination function of the Sequence Detection System V.2.4 (Applied Biosystems, Waltham, MA, USA).

### Neuropsychiatric Assessment

All participants completed standardized, comprehensive neuropsychological assessments of seven cognitive domains that are commonly affected by HIV (learning, recall, motor function, executive function, working memory, speed of information processing, and verbal fluency) (Heaton et al. 2010). The best available normative standards were corrected for the effects of age, education, sex, and race/ethnicity, as described previously (Cysique et al. 2011). Test scores were converted to demographically corrected standard T scores, which were then converted into deficit scores. The deficit scores across the entre battery were then averaged to derive a Global Deficit Score (GDS), which ranges from 0 to 5, with higher scores indicating poorer NC performance (Carey et al. 2004). A GDS value ≥ 0.5 indicates NCI. Depressive symptoms were assessed by the Beck Depression Inventory Second Edition (BDI-II), a validated survey of 21 questions that assess the intensity, severity and depth of depressive symptoms (Beck et al. 1996). Higher BDI-II values indicate more depressive symptoms with a value > 13 indicating at least mild depression. Minimal comorbidities were defined based on the guidelines by Antinori et al. (Antinori et al. 2007), and concordance analysis was performed to confirm that there was good agreement on the classification of study participants with respect to this system.

### Statistical Analysis

To assess explanatory variables for impaired vs. unimpaired participants in the cohorts, categorical data were compared using Pearson’s chi-square test, and continuous data were compared using Student’s *t* test for normally distributed variables and the Mann-Whitney U test for nonnormally distributed variables, after determining the distribution using the Shapiro-Wilk test. Univariable and multivariable methods, including logistic and linear regression, were used to determine the association of the noncoding and coding SNVs with demographic characteristics (e.g., age, sex, and ancestry), disease-related variables (e.g., plasma and cerebrospinal fluid (CSF) HIV RNA, nadir CD4^+^ T cell count, comorbidity (e.g., depression, HCV coinfection), treatment status (e.g., ART use), and NC performance, including global deficit score (GDS), global NCI, and domain-specific deficit scores (DDSs). To account for type I error in the analyses of multiple variables associated with GDS and NCI, the false discovery rate approach was used. Akaike Information Criterion and backward selection were used in multivariable regression. All statistical analyses were performed using JMP Pro version 15.0 (SAS Institute, Cary, NC, USA).

## Results

### The Characteristics of the Participants

Table 2 summarizes the characteristics of the 1047 individuals who had data on noncoding *EIF2AK3* SNVs. Briefly, the mean age was 43.1 years, 22.8% of the cohort were female, and 41% had European ancestry. Most participants had AIDS (60.7%) and were categorized as having only “minimal” neuropsychiatric comorbidities (64.1%). The median CD4^+^ T cell counts were 175/µL (nadir) and 428/µL (current). At the first assessment, 70.5% used ART and 68.3% of these had plasma HIV RNA levels ≤ 200 copies/mL.

**Table 2:** Characteristics of the participants according to the neurocognitive impairment status

	All (*n* = 1047)	Unimpaired (*n* = 663)	Impaired (*n* = 384)	*p*
Age, y, mean	43.1	43.0	43.4	0.26
Sex, female	22.8%	24.2%	22.1%	0.456
Ancestry				**0.002**
European ancestry	41.0%	40.8%	41.4%	0.84
Admixed Hispanic	12.3%	9.9%	17.0%	**0.001**
African ancestry	46.7%	49.3%	41.7%	**0.003**
NP comorbidity	64.1%	70.4%	53.1%	**< 0.001**
AIDS diagnosis	60.7%	58.2%	65.6%	**0.021**
HCV seropositivity	25.9%	26.1%	24.2%	0.36
Nadir CD4^+^ T cell count (/µL), median (IQR)	175 (50–300)	190 (54–320)	150 (36–257)	**0.028**
Current CD4^+^T Cells (/µL), median (IQR) (*n* = 1040)	428 (265–600)	426 (269–593)	434 (250–607)	0.97
ART – current use	70.5%	67.4%	76.4%	**0.002**
Plasma HIV RNA ≤ 200 copies/mL (on ART)	68.3%	69.2%	66.3%	0.41

*Abbreviations* *AIDS* acquired immunodeficiency syndrome; *IQR* interquartile range; *NP* neuropsychiatric

Consistent with prior reports from CHARTER, analyses identified that NCI (*n* = 384, 37.4%) was associated with AIDS (65.6% vs. 58.2% in the 663 participants without NCI, *p* = 0.021), ART use (76.4% vs. 67.4%, *p* = 0.002), and lower nadir CD4^+^ T cell count (150 vs. 190/µL, *p* = 0.028), and more than minimal comorbidities (70.4% vs. 53.1%, *p* < 0.001).

Regarding the noncoding SNVs, 41.7% of the cohort had at least one T minor allele for rs6739095, 41.4% of the cohort had at least one C minor allele for rs1913671, and 39.4% of the cohort had at least one C minor allele for rs11684404. The frequencies of the minor alleles of the three noncoding SNVs reflected those reported in the general population (Online Resource 1). The concordance among the three minor alleles of the three noncoding SNVs was also high in the CHARTER cohort (Online Resource 2), in agreement with previous GWAS findings.

### Minor Alleles of Noncoding *EIF2AK3* SNVs are Associated with Worse GDS and NCI

**Fig. 1:**
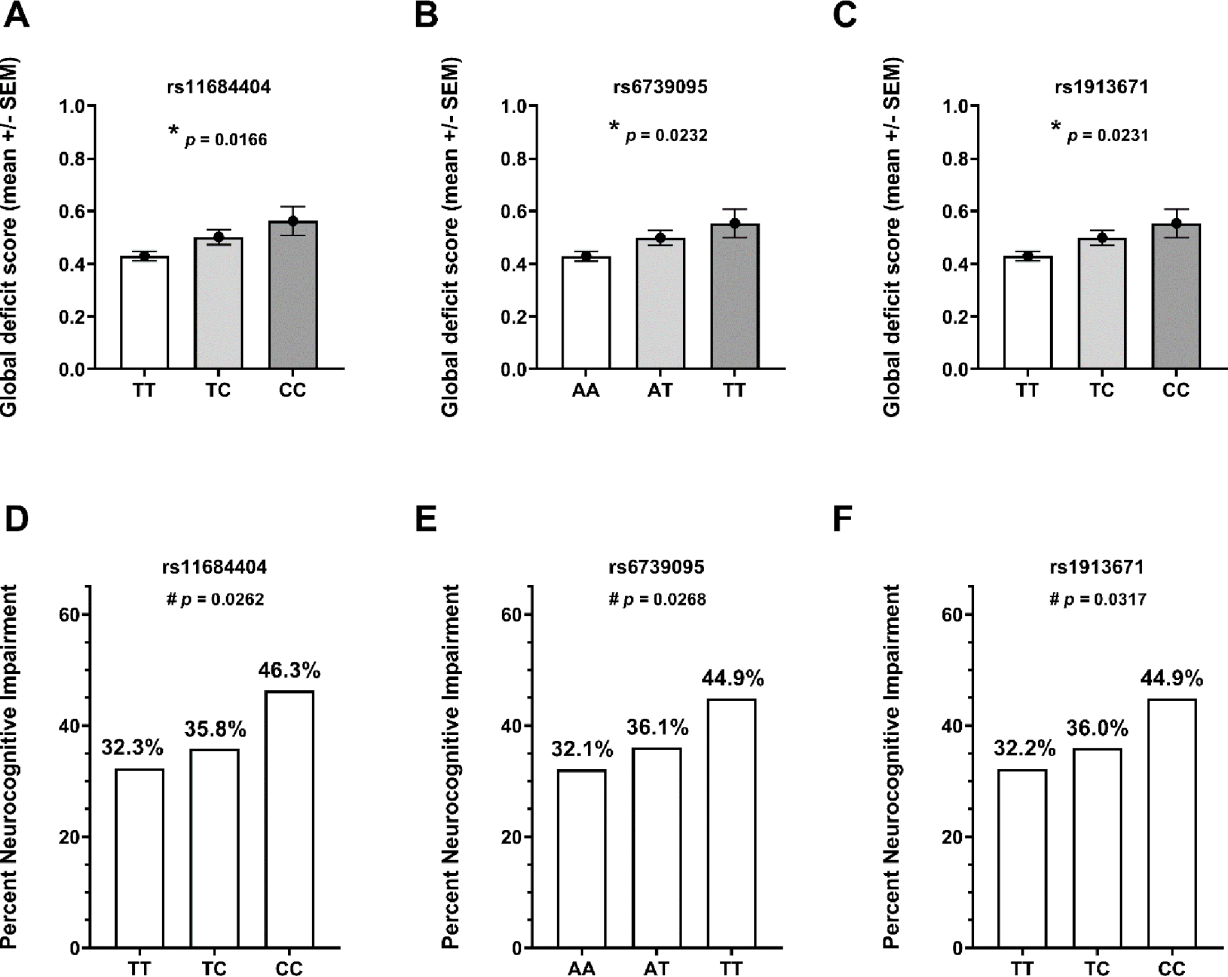
Association of noncoding *EIF2AK3* SNVs with GDS (**A**–**C**) and NCI (**D**–**E**) in the GWAS cohort. *Abbreviations GDS* global deficit score; *NIC* neurocognitive impairment; *SNV* single nucleotide variant. * *p* < 0.05, analysis of variance; ^#^*p* < 0.05, Cochran–Armitage test

We first examined the association of noncoding *EIF2AK3* SNVs with GDS in the GWAS dataset. As shown in Fig. 1, we found that the minor alleles of all three noncoding SNVs were associated with worse GDS and NCI in a dose-dependent manner. For example, the mean GDS values were 0.43 (TT), 0.50 (CT), and 0.56 (CC) for the rs11684404 C risk allele (Fig. 1A, *p* = 0.0016). The frequency of NCI was also significantly higher in CC homozygotes than in TT homozygotes for rs11684404 (Figs. 1D and 46.3% vs. 32.3%, *p* = 0.0262). Similarly, the rs6739095 T and rs1913671 C risk alleles were associated with GDS as well as with NCI (Fig. 1B, 1 C, 1E, 1 F).

### Minor Alleles of Noncoding *EIF2AK3* SNVs are Associated with Performance in Specific NC Ability Domains

Emerging data from recent studies including CHARTER and the MACS/WIHS Combined Cohort Study (MWCCS) support that several indicators such as sex and inflammation are associated with deficits in specific NC domains, such as delayed recall or working memory (Dastgheyb et al. 2019; Rubin et al. 2020). We analyzed the associations of the three noncoding *EIF2AK3* SNVs with deficits in NC domains in the CHARTER GWAS dataset. As shown in Table 3, the minor alleles of all three noncoding SNVs were associated with deficits in executive functioning (*p* < 0.001), verbal fluency (*p* < 0.05), motor function (*p* < 0.05), and possibly learning (p values, 0.038 to 0.054).

**Table 3:** Association of noncoding *EIF2AK3* SNVs with domain-specific scores

Domain-specific T score	*p* value		
	rs6739095	rs1913671	rs11684404
Verbal	**0.0256**	**0.0198**	**0.0103**
Executive	**< 0.001**	**< 0.001**	**< 0.001**
Motor	**0.0117**	**0.0230**	**0.0138**
Learning	**0.0542**	**0.0524**	**0.0381**
Recall	0.7697	0.7779	0.9020
Working memory	0.3534	0.3962	0.4429
Speed of information processing	0.354	0.350	0.336

### Minor Alleles of Noncoding *EIF2AK3* SNVs are Independently Associated with GDS and NCI

Multiple linear regression analysis was conducted to explore the association of the noncoding *EIF2AK3* SNVs and the HIV and host factors previously shown to be related to GDS. The analysis revealed that the minor allele of rs11684404 remained significantly associated with GDS, along with ART use and neuropsychiatric comorbidities (*R*^*2*^ = 0.08, *p* < 0.0001) (Table 4), whereas the minor alleles of the other two noncoding *EIF2AK3* SNVs did not remain significantly associated with GDS after accounting for the influence of rs11684404. In contrast, multiple logistic regression analysis identified that NCI was associated with the minor allele of rs6739095, but not rs11684404, as well as with ART use, neuropsychiatric comorbidities, and HCV seropositivity (*R*^*2*^ = 0.04, *p* < 0.0001) (Table 4)

**Table 4:** Multivariable analysis of the CHARTER dataset

	Univariable		Multivariable	
	β	*p* value	β	*p* value
GDS (Model R^2^ = 0.080, *p* = 2.1 × 10^− 16^)				
rs6739095, T	0.08	**0.0058**		
rs11684404, C	0.083	**0.0026**	0.048	**0.0017**
Neuropsychiatric Comorbidities	0.111	**< 0.001**	0.111	**< 0.001**
ART Use	0.058	**< 0.001**	0.038	**0.047**
Nadir CD4^+^ T-Cells	−2.5 × 10^− 4^	**0.0018**	−1.7 × 10^− 4^	**0.059**
AIDS diagnosis	0.042	**0.006**		
BDI-II > 13	0.041	**0.012**	0.023	**0.153**
HIV RNA log_10_ c/mL	−0.027	**0.021**		
Age	0.003	**0.077**		
Ancestry	−0.026	**0.100**	−0.027	**0.085**
**NCI (Model R**^**2**^ **= 0.044, p = 1.1 × 10**^**− 11**^**)**				
rs6739095, T	0.285	**0.024**	0.164	**0.015**
rs11684404, C	0.299	**0.018**		
Neuropsychiatric Comorbidities	0.371	**< 0.001**	0.401	**< 0.001**
ART Use	0.267	**< 0.001**	0.279	**< 0.001**
Nadir CD4^+^ T-Cells	0.001	**0.0046**		
BDI-II > 13	0.163	**0.018**	0.104	**0.148**
AIDS diagnosis	0.142	**0.032**		
HCV serostatus	−0.126	**0.096**	−0.221	**0.005**

Candidate covariates tested included demographic (e.g., age, race, sex), HIV disease (e.g., HIV RNA in plasma, nadir and current CD4^+^ T cell counts), HIV treatment (e.g., ART use), and common comorbidities (e.g., neuropsychiatric comorbidities, HCV co-infection). The *p* values for BDI-II > 13 are greater than 0.05 but the covariate was retained in the model by Akaike Information Criterion selection

### Minor Alleles of Coding *EIF2AK3* SNVs are Associated with Worse GDS and NCI

**Fig. 2:**
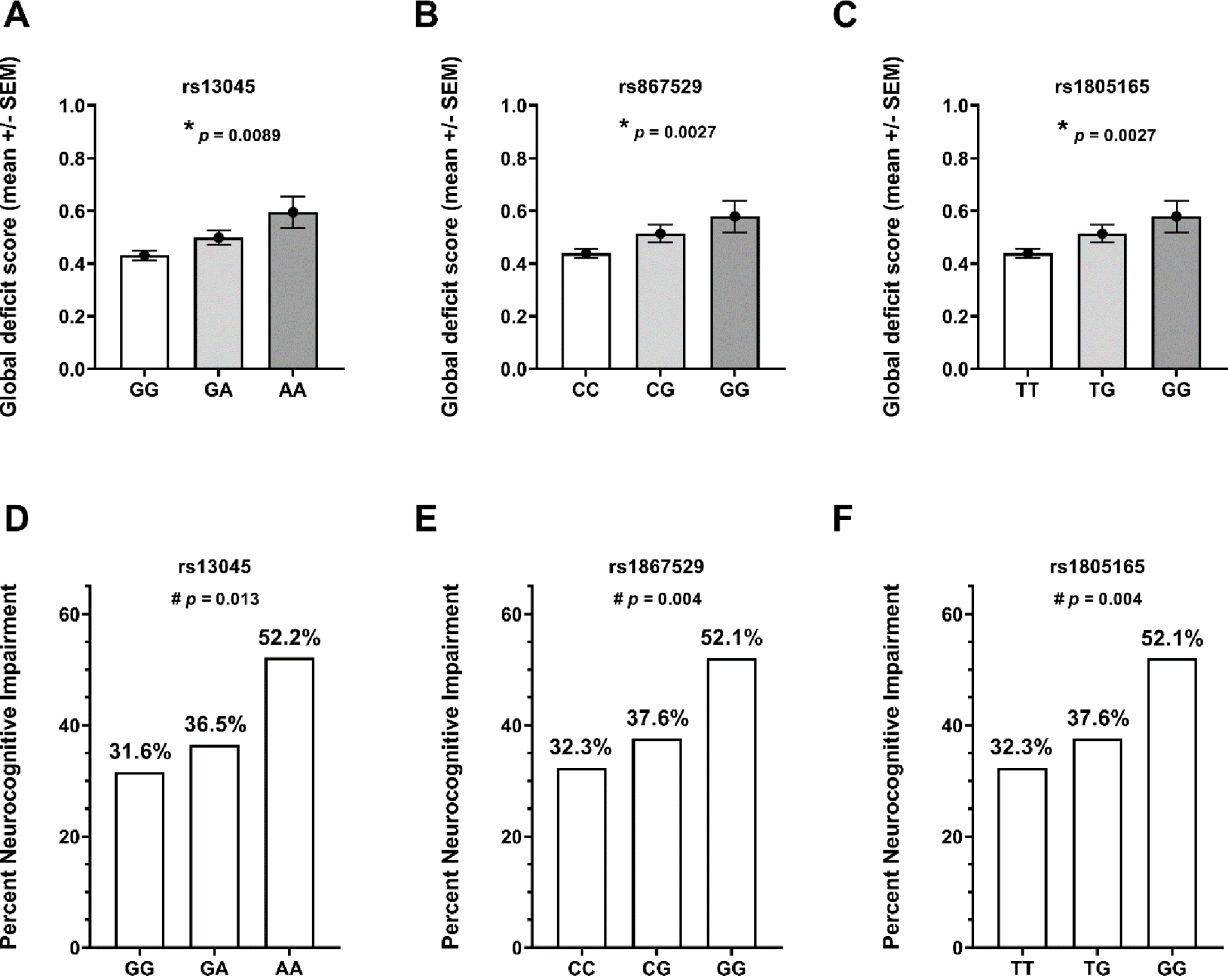
Association of coding *EIF2AK3* SNVs with GDS (**A**–**C**) and NCI (**D**–**E**) in the TS cohort. * *p* < 0.05, analysis of variance; ^#^*p* < 0.05, Cochran–Armitage test

Based on our analyses indicating associations of noncoding *EIF2AK3* SNVs with GDS and NCI, we next analyzed the minor alleles of three coding *EIF2AK3* SNVs, which were previously shown to be associated with increased risk of PSP(Hoglinger et al. 2011; Stutzbach et al. 2013). The characteristics of this TS sub-cohort, which included 992 participants with available genomic DNA for analysis, are summarized in Online Resource 3. Risk alleles were again present in a substantial minority: at least one A allele for rs13045 in 41.3%, at least one G allele for rs867529 in 30.9%, and at least one G allele for rs1805165 in 30.9%. The frequencies of the minor alleles of these three SNVs are consistent with their reported frequencies across the 26 published GWASs (Online Resource 4). However, the concordance among the three coding SNVs was different than that observed among the three noncoding SNVs. Specifically, rs867529 and rs1805165 were 100% concordant whereas each exhibited 88.3% concordance with rs13045 (Online Resource 5). Even with this difference in concordance, the minor alleles of all three coding SNVs were also significantly associated with GDS and NCI in a dose-dependent manner, as shown in Fig. 2.

### Minor Alleles of Coding *EIF2AK3* SNVs are Associated with Deficits in Cognitive Domains

Our analyses to investigate the associations of the three coding *EIF2AK3* SNVs with deficits in specific NC domains in the TS cohort revealed distinct patterns, as shown in Table 5. Specifically, the minor allele of rs13045 was associated with domain-specific deficits in executive functioning (*p* = 0.0131), learning (*p* = 0.0213), and motor domains (*p* = 0.0116) whereas the minor alleles of rs867529 and rs1805165 were significantly associated with domain-specific deficits in verbal fluency (*p* = 0.0025) and motor domains (*p* = 0.0078).

**Table 5:** Association of coding *EIF2AK3* SNVs with domain-specific scores

Domain-specific T score	*p* value		
	rs13045	rs867529	rs1805165
Motor	**0.0116**	**0.0078**	**0.0078**
Verbal	**0.081**	**0.0025**	**0.0025**
Executive	**0.0131**	**0.0518**	**0.0518**
Learning	**0.0213**	0.1259	0.1259
Recall	0.1368	0.0650	0.0650
Working memory	0.5629	0.1032	0.1032
Speed of information processing	0.229	0.358	0.358

### Minor Allele of rs13045 is Independently Associated with GDS and NCI

Our multiple logistic regression analysis comparing the coding *EIF2AK3* SNVs to HIV and host factors revealed that the minor allele of rs13045 remained significantly associated with NCI, along with ART use and a BDI-II score > 13 (Beck et al. 1996; Ellis et al. 2020) (*R*^*2*^ = 0.048, *p* < 0.0001) (Table 6), although the minor alleles of the other two coding *EIF2AK3* SNVs did not. Multiple linear regression of GDS confirmed this finding: only the rs13045 minor allele (but not the other coding SNVs) remained associated with GDS, along with nadir CD^+^ T-cell count, BDI-II > 13, and European ancestry (*R*^*2*^ = 0.03, *p* < 0.0001) (Table 6).

**Table 6:** Multivariable analysis of the targeted sequencing sub-cohort for NCI and GDS

GDS (Model R^2^ = 0.03, *p* < 0.0001)					
	Risk direction	β	*p* value	β	*p* value
rs13045	A allele	0.076	**0.002**	**0.057**	**0.025**
Neuropsychiatric comorbidities	Yes	0.074	**0.017**		
ART Use	Current use	0.072	**0.041**		
Nadir CD4^+^ T-Cells	Lower	−1.7 × 10^− 4^	**0.045**	**−1.7 × 10** ^**− 4**^	**0.048**
AIDS diagnosis	Yes	0.076	**0.016**		
BDI-II > 13	> 13	0.107	**< 0.001**	**0.096**	**0.002**
HIV RNA log_10_ c/mL	Lower	−0.022	0.07		
Age	Older	0.003	0.141		
Ancestry	European	0.111	**< 0.001**	**0.093**	**0.004**
**NCI (Model R**^**2**^ **= 0.048, p < 0.0001)**					
rs13045	A allele	0.338	**0.015**	0.339	**0.003**
Neuropsychiatric comorbidities	Yes	0.28	**0.041**		
ART Use	Current use	0.530	**0.001**	0.504	**0.002**
Nadir CD4^+^ T-Cells	Lower	−6 × 10^− 4^	0.14		
AIDS diagnosis	Yes	0.19	0.17		
BDI-II > 13	> 13	0.539	**< 0.001**	0.534	**< 0.001**
HIV RNA log_10_ c/mL	Lower	−0.117	**0.031**		
Age	Older	0.006	0.44		
Ancestry	European	0.342	**0.012**		

### Minor Alleles of Coding *EIF2AK3* SNVs are Associated with Increased CSF Levels of IL-6

**Fig. 3:**
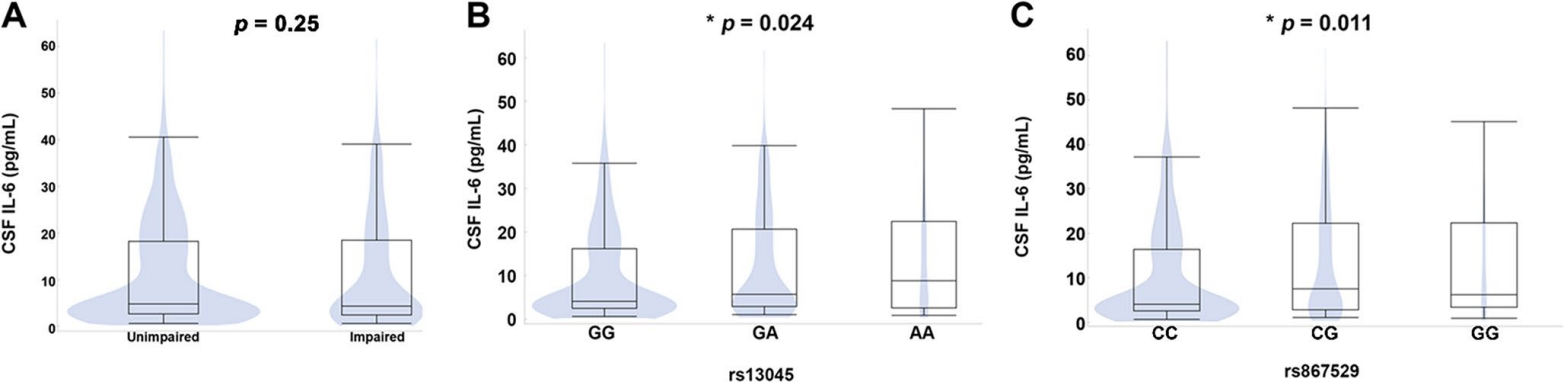
Association of NCI (**A**) and two coding *EIF2AK3* SNVs (**B**, **C**) with IL-6 in CSF in a subset of the study cohort with available data. * *p* < 0.05, analysis of variance

Based on studies finding that IL-6 concentrations in blood and CSF are a biomarker of inflammation that is associated with NCI in PWH (Oliveira et al. 2017), we assessed the relationship of the coding *EIF2AK3* SNVs with this biomarker in participants with CSF IL-6 concentrations that were previously quantified by immunoassay and stored in the CHARTER data repository (*n* = 534). The CSF IL-6 levels did not significantly differ between those with and without NC impairment (Fig. 3A). The minor alleles of all three coding *EIF2AK3* SNVs were significantly associated with higher IL-6 in CSF (Fig. 3B, 3 C; rs13045: *p* = 0.024, rs867529: *p* = 0.011, and rs1805165: *p* = 0.011; data not shown for rs1805165). In contrast, the minor alleles of these coding SNVs were not associated with IL-6 concentration in blood plasma among participants with available data (*n* = 144; data not shown).

### Association of *EIF2AK3* Haplotypes with NCI and GDS

Based on the complete concordance between rs867529 and rs1805165, we constructed *EIF2AK3* haplotypes in the TS cohort. The frequency of these haplotypes were *EIF2AK3* A/A 58.7% (*n* = 583), A/B 24.7% (*n* = 245), A/D 9.8% (*n* = 97), B/B 4.8% (*n* = 48), B/D 1.4% (*n* = 14), and D/D 0.5% (*n* = 5) (Online Resource 6). The haplotype groups did not differ in participant characteristics. *EIF2AK3* haplotypes were not associated with NCI or GDS (data not shown) or with IL-6 concentrations in CSF (Online Resource 7).

## Discussion

One of the conundrums of the ART era is the persistence of NCI even in PWH who have achieved viral suppression and immune recovery with ART. Up to 32.7% of PWH exhibit NCI-related dysfunction in activities of daily living and even asymptomatic NCI in PWH confers substantial risk for transitioning to symptomatic status over a period of just a few years (Grant et al. 2014; Heaton et al. 2015; Wei et al. 2020). In this retrospective analysis of the well characterized CHARTER cohort, we found that previously defined coding and noncoding *EIF2AK3* minor risk alleles were significantly associated with global NCI and the continuous GDS impairment measure. We also observed that the minor alleles of all three coding *EIF2AK3* SNVs were associated with higher IL-6 concentrations in CSF. Furthermore, the associations between the rs13045 A minor risk allele and either global NCI or GDS remained significant after adjustment for other covariates in multivariable models. The three coding *EIF2AK3* minor risk alleles were associated with performance in four NC domains: motor functioning, verbal fluency, executive functioning, and learning. While recent studies have reported certain coding and noncoding *EIF2AK3* SNVs as genetic risk factors in several neurodegenerative diseases, including AD and PSP (Hoglinger et al. 2011; Stutzbach et al. 2013), this is the first study to demonstrate an association between multiple coding and noncoding *EIF2AK3* SNVs and cognition in PWH. Following our previous studies where we show increased expression of phospho-PERK and peIF2α in the neurons in the midfrontal cortical tissue of ART-treated patients with HIV-associated neurocognitive impairment (Akay et al. 2012), these findings support accumulating evidence of ISR activation as a contributor to the pathogenesis of HIV in the CNS (Akay et al. 2014; Bond et al. 2020; Fan and He 2016; Gannon et al. 2017; Ma et al. 2016; Roth et al. 2021; Stern et al. 2017) and implicate *EIF2AK3* as a potentially important component of genetic vulnerability to NCI in PWH.

Among the four major *EIF2AK3* haplotypes, haplotypes A and B encode protein products that differ by changes in two amino acids in the luminal domain (S136C, R166Q) and one amino acid in the kinase domain (S704A) of PERK (Table 1). The significant associations of minor risk alleles of *EIF2AK3* coding SNVs with NC performance uncovered in the present study are likely related to differences in PERK activation, activity, or downregulation resulting from these amino acid changes. Importantly, PERK signaling through phosphorylation of translation initiation factor, eIF2α, and antioxidant response transcriptional regulator, NRF2, is linked to neuronal, astrocytic, and macrophage/microglial function, health, and stress response (Guthrie et al. 2016; Meares et al. 2014; Moreno et al. 2013; Raines et al. 2022; Sanchez et al. 2019; Stone et al. 2019). Tight regulation of PERK kinase activity to a narrow window is necessary for the resolution of stress without pathologic effects: Constant low-level non-lethal ER stress preconditions and provides acquired resilience against endogenous and exogenous stresses by priming downstream signaling cascades such as antioxidant response and autophagy (Green 2021; Halliday et al. 2017; Rabouw et al. 2019; Urra et al. 2013). Therefore, differential stress response and tolerance of the different PERK haplotypes might contribute to our observed association of minor alleles of *EIF2AK3* SNVs with neurocognitive impairment.

However, the impact of these *EIF2AK3* SNVs on PERK kinase activity and downstream cellular events remains unclear due to contradictory findings reported by a limited number of studies. In one study, lymphoblastoid cell lines derived from *EIF2AK3* haplotype B-expressing individuals, which exhibited similar baseline PERK protein levels, nonetheless displayed enhanced PERK kinase activity, as determined by peIF2α accumulation, in response to challenge with tunicamycin, which blocks N-linked glycosylation and induces ER stress (Liu et al. 2012). In contrast, another study proposed that *EIF2AK3* haplotype B might be a hypomorph based on higher eIF2α phosphorylation in *EIF2AK3* haplotype A-expressing cells compared to *EIF2AK3* haplotype B-expressing cells, both at baseline and following treatment with thapsigargin, which inhibits the sarcoplasmic/ER Ca2^+^ ATPase pump. The association of specific *EIF2AK3* SNVs with increased kinase activity and deleterious outcomes is likely cell- and context-dependent. Nonetheless, our analyses showing the significant association of the minor alleles of the three coding *EIF2AK3* SNVs with NC performance corroborate our previous studies demonstrating ISR activation in the post-mortem brain tissue from ART-treated PWH (Akay et al. 2012) and implicate that maladaptive signaling might be at play in the CNS of PWH harboring the minor alleles of *EIF2AK3* SNVs.

In the TS analysis, only the rs13045 A allele remained significantly associated with both GDS and NCI in multivariable analyses. The rs13045 minor allele is present in both haplotypes B and D, whereas haplotype D is defined by the presence of the rs13045 A allele alone. The frequency of the rs13045 A allele is higher than the frequencies of the other two coding *EIF2AK3* SNVs across different racial groups around the globe (Online Resource 8), which might partially explain the lack of association of the other two SNVs in multivariable analysis in the present study. The number of haplotype D heterozygotes or homozygotes was small (116/992), precluding more in-depth analysis. In addition to the need for follow-up studies in larger cohorts to clarify whether haplotype D confers increased risk for NCI, mechanistic studies are also warranted to determine whether the change in amino acid 166 from arginine to glutamine in cells carrying the rs13045 A allele may facilitate weaker affinity for binding immunoglobulin protein (BiP), allowing its dissociation from PERK, with subsequent reduction in PERK kinase activation threshold

Our multivariable analyses of the TS cohort also confirmed that a level of depressive symptoms consistent with at least mild depression (BDI-II > 13) was also significantly associated with NC performance, supporting the contribution of depression to worse NC outcomes in PWH, which has been recently reported in the CHARTER cohort (Saloner et al. 2021). Adding to the findings of previous studies reporting the association of depression with genotype and cognitive function in PWH (Avdoshina et al. 2013; Levine et al. 2009), these results also implicate a potential interaction between the rs13045 A allele and depression, which is currently under investigation. As the most common neuropsychiatric comorbidity in PWH (REF), depression is associated with worse ART adherence, viral suppression, and survival (Ickovics et al. 2001; Ironson et al. 2005; Villes et al. 2007). Although no study to date has reported a link between *EIF2AK3* SNVs and depression, the expression levels of several UPR-related genes and proteins, including *EIF2AK3* and PERK, are higher in adults with major depressive disorder (Bown et al. 2000; Nevell et al. 2014). In addition, depressive-like behaviors and memory impairment can be alleviated by inhibiting PERK-peIF2α signaling in animal models of depression (Li et al. 2019; Sharma et al. 2018)

The observed association between an *EIF2AK3* SNV and higher IL-6 concentrations in CSF is particularly interesting since several studies have linked systemic and CNS inflammation to neuropsychiatric conditions, including worse NC performance and depressive symptoms (Ellis et al. 2020; Sattler et al. 2015). This finding provides additional evidence for a potential role of PERK in neuroinflammation in several disorders including NCI in PWH (Meares et al. 2014; Roth et al. 2021)

In the present study, we found that ART use was independently associated with neurocognitive impairment, in agreement with an earlier CHARTER cohort study (Heaton et al. 2010). Starting in mid-2000s, several studies suggested that antiretroviral drugs might be associated with poorer cognitive functioning (Heaton et al. 2010; Joska et al. 2010; Marra et al. 2009; Robertson et al. 2010). This finding can be explained by more advanced disease requiring ART, failure of viral suppression despite ART, and potential antiretroviral drug toxicity. To this last point, in several in vitro and in vivo studies, we previously demonstrated that certain antiretroviral drugs from specific classes led to damage and dysfunction in several cell types in the CNS, including neurons and oligodendrocytes (Akay et al. 2014; Gannon et al. 2017; Roth et al. 2021; Stern et al. 2017). For example, in a recent study, we demonstrate within-class as well as cross-class differences in antiretroviral drug-mediated neuronal damage, which includes the ISR (Stern et al. 2017). Further investigation is warranted to assess whether *EIF2AK3* SNVs are associated with adverse effects of specific antiretroviral drugs in the CNS.

The CHARTER cohort includes PWH with long-term HIV, raising the possibility that our findings might reflect the legacy effect of HIV infection. The current cohort of PWH entered the CHARTER cohort between 2003 and 2007 and the participants were considered to be already at higher risk of cognitive impairment due to the ART initiation guidelines in place at that time, which were based on low CD4^+^ T cell counts. Therefore, it is possible that delayed initiation of ART could have contributed to poorer cognitive function. While we did not find an association between nadir CD4^+^ cell count < 200/µL and any of the coding or noncoding *EIF2AK3* SNVs, studies with larger cohorts of PWH who were initiated on ART soon after infection are necessary to assess the potential legacy effect

The significant association of the minor alleles of specific *EIF2AK3* SNVs with deficits in specific domains raise the possibility of a link between specific SNVs and certain NC phenotypes, including declines in verbal fluency, executive function, learning, and motor function, which have been recently described in the MWCCS (Dastgheyb et al. 2019; Molsberry et al. 2018; Rubin et al. 2017). These phenotypes likely differ in viral, host, and genetic mechanisms. Importantly, in a recent study by May et al.(May et al. 2020), factor analysis of the CHARTER cohort revealed that individual neuropsychological tests did not load exactly onto the expected domains that were included in our study, suggesting the need for another framework for future analyses of cognitive domains before further analyses can be conducted to examine potential latent cognitive factors contributing to the observed association of minor alleles of specific *EIF2AK3* SNVs with deficits in specific domains. Nonetheless, Kolson and colleagues recently reported associations between regional brain neuroinflammation and heme oxygenase-1 (HO-1) responses in analyses that included brain tissue from autopsy and CSF from PWH taking ART (Gill et al. 2018). Their findings also implicate regional HO-1 isoform expression may be associated with differences in recovery from acute synaptic injury in a primate model of lentiviral infection (Garcia-Mesa et al. 2020). Given the well-characterized mechanistic role of PERK and HO-1 in activating the antioxidant response (Cullinan and Diehl 2004), an interaction between *EIF2AK3* SNVs and regional susceptibility to oxidative stress might partially contribute to the observed associations of specific minor alleles of *EIF2AK3* SNVs with NC phenotypes

The construction of the *EIF2AK3* haplotypes in the TS cohort revealed that the frequencies of specific haplotypes were similar to those reported in the general population, supporting that our findings may be generalizable (Online Resource 6). The comparison of domain-specific deficit scores among groups categorized according to haplotypes suggest that certain haplotypes might be associated with deficits in certain domains (Online Resource 9); however, the number of participants in certain groups was low, precluding further statistical analyses. Future studies are warranted in other, larger cohorts such as the MWCCS, which has notable differences including greater inclusion of women and people without HIV as well as a different distribution of ancestry, to determine the potential role of *EIF2AK3* as a genetic risk factor for NCI in PWH.

Our retrospective analysis of the CHARTER GWAS data revealed that the minor alleles of the three noncoding *EIF2AK3* SNVs, which were in strong linkage disequilibrium with those of the three coding *EIF2AK3* SNVs, were also significantly associated with GDS and NCI. The impact of these noncoding SNVs on PERK expression, function or stability is unclear, although they have previously been linked to increased risk for PSP (Stutzbach et al. 2013). All three noncoding SNVs are located in the intronic regions of *EIF2AK3* and may result in the formation of alternative transcripts leading to RNA species or protein isoforms with altered stability or function, which warrants further investigation

In light of the evidence showing increased or sustained PERK activity as detrimental to several critical cellular pathways in a wide range of conditions (Devi and Ohno 2014; Salminen et al. 2009; Zhang and Kaufman 2008), we propose that minor alleles of SNVs associated with increased *EIF2AK3* activity in response to stress might be a predictive risk factor for NCI in PWH. Aging-related comorbid diseases may cause sustained ISR activation via PERK (Devi and Ohno 2014; Gannon et al. 2017; Stern et al. 2018) that may further increase the risk for NCI in PWH.

Our findings raise several fundamental questions that should be addressed before establishing *EIF2AK3* as a risk factor for NCI in PWH. First, the regulation and kinase activity of disease-associated *EIF2AK3* SNVs should be described. Second, mechanisms underlying the association of specific *EIF2AK3* SNVs with deleterious cellular responses to disease-relevant stressors should be elucidated in pertinent cellular contexts. Furthermore, whether the observed association of these SNVs with specific domains can be replicated and used to reliably identify specific NC phenotypes should be clarified. Future studies should also explore the relationship of *EIF2AK3* SNVs with molecular and imaging biomarkers of NCI in PWH. Additionally, elucidation of the interrelationships between *EIF2AK3* SNVs and previously proposed genetic factors may potentially lead to the development of a polygenic risk score as part of a precision medicine approach to identify phenotypes and specific populations who may specifically benefit from adjunctive therapies such as modulators of PERK and endogenous antioxidant response during the best therapeutic window, soon after HIV diagnosis

The present study has several limitations that should be addressed in future studies. First, the study cohort was relatively small for a genetics study. However, the distributions of both noncoding and coding *EIF2AK3* SNVs in the CHARTER cohort were similar to those reported in larger GWASs (Stutzbach et al. 2013). Additionally, the concordance among the noncoding and coding *EIF2AK3* SNVs followed the expected pattern reported in the general population. Second, the CHARTER cohort does not include healthy individuals and may raise the question of whether our findings reflect HIV-related pathophysiology. The frequencies of minor *EIF2AK3* alleles examined in this study range between 15–30% in the general population across different ancestries. While several studies reported the association of some of these *EIF2AK3* SNVs with neurodegenerative conditions, the associations appear to be relatively minor, suggesting that these SNVs may not be critical drivers of cognitive weaknesses in the general population. Additionally, ISR activation, including the activity of PERK, must be tightly regulated to an optimal level for successful stress response without pathological effects, given that ISR activation aims to boost fundamental biological responses to resolve multi-organellar stress, metabolic function, and protein quality control. In PWH harboring specific *EIF2AK3* SNVs, a wide range of host and viral factors can affect PERK activity and regulation and even subtle changes in the regulation or activity deviating from an optimal range can have detrimental outcomes during the chronic disease course. In other words, these *EIF2AK3* SNVs may not matter in the absence of lifelong chronic stress. Therefore, we predict that these SNVs are less likely to be problematic in the general population, while putting people with HIV at greater risk and that our findings are likely an indication of the underlying pathologic processes specifically observed in PWH and not in HIV-uninfected individuals. Third, due to the low number of cases with some haplotypes, we were unable to examine the relationship of *EIF2AK3* haplotypes with CSF IL-6 concentrations or BDI-II scores. The cross-sectional design precludes the inference of a causal relationship between *EIF2AK3* haplotypes and NC impairment. Studies in animal models are warranted to reveal the mechanistic underpinnings of the observed associations. Finally, the models did not explain substantial variance in GDS or NCI. While this is true, the effect size for *EIF2AK3* SNVs was similar in magnitude to effect sizes attributable to ART use, neuropsychiatric comorbidities, and other covariates, highlighting the potential importance of this genetic vulnerability

## Conclusions

Minor alleles of coding and non-coding SNVs of *EIF2AK3* were associated with global NC and domain-specific performance. These associations, albeit small-to-medium in size, were present in multivariable analyses, suggesting that specific SNVs in *EIF2AK3*, and perhaps other ISR-related genes, may be a component of genetic vulnerability to cognitive, motor and/or behavioral dysfunction in PWH. Determination of host factors that are predictive for NC phenotypes early in the course of HIV disease might enable more effective preventive and therapeutic interventions for NC disorders.

## Electronic Supplementary Material

Below is the link to the electronic supplementary material.

Supplementary Material 1

## Data Availability

No datasets were generated or analysed during the current study.
