# Supplementary material for: Genetic Variations in EIF2AK3 are Associated with Neurocognitive Impairment in People Living with HIV
Source: J Neuroimmune Pharmacol. 2024 May 25;19(1):25. doi: 10.1007/s11481-024-10125-x (PMC11126443; doi:10.1007/s11481-024-10125-x)
Supplement: Supplementary file 1 — Supplementary Material 1 [file 11481_2024_10125_MOESM1_ESM.docx]

**Supplementary Material**

**Genetic variations in *EIF2AK3* are associated with neurocognitive impairment in people living with HIV**

Cagla Akay-Espinoza, Sarah E.B. Newton, Beth A. Dombroski, Asha Kallianpur, Ajay Bharti, Donald R. Franklin, Gerard D. Schellenberg, Robert K. Heaton, Igor Grant, Ronald J. Ellis, Scott L. Letendre, Kelly L. Jordan-Sciutto

**Corresponding author:**

Kelly Jordan-Sciutto

Department of Oral Medicine, School of Dental Medicine, University of Pennsylvania

240 S. 40th St, Rm 312 Levy, Philadelphia, PA 19104

Phone: 215-898-4196; E-mail: jordank@upenn.edu

**Online Resource 1.** Minor allele frequencies of noncoding *EIF2AK3* SNVs

|  | Number of minor alleles | | |
| --- | --- | --- | --- |
|  | 0 | 1 | 2 |
| rs6739095, T | 58.3% | 35.1% | 6.6% |
| rs1913671, C | 58.3% | 34.8% | 6.6% |
| rs11684404, C | 60.6% | 33.0% | 6.4% |

SNV, single nucleotide variant

**Online Resource 2.** Concordance of noncoding *EIF2AK3* SNVs

|  | rs6739095 | rs1913671 | rs11684404 |
| --- | --- | --- | --- |
| rs6739095 | - |  |  |
| rs1913671 | 99.6% | - |  |
| rs11684404 | 96.8% | 96.9% | - |

SNV, single nucleotide variant **Online Resource 3.** Characteristics of the targeted sequencing sub-cohort of participants included in targeted sequencing

|  | All  (n = 992) | Unimpaired  (n = 636) | Impaired  (n = 336) | *p* |
| --- | --- | --- | --- | --- |
| Age, y (mean) | 43.7 | 44.0 | 43.6 | 0.27 |
| Sex, female | 22.6% | 22.7% | 22.4% | 0.93 |
| Ancestry |  |  |  | <0.0001 |
| European | 42.2% | 39.5% | 47.5% |  |
| Admixed Hispanic | 9.8% | 6.6% | 15.7% | 0.004 |
| African ancestry | 45.9% | 52.3% | 33.8% | <0.001 |
| AIDS diagnosis | 61.9% | 60.3% | 64.7% | 0.18 |
| HCV seropositivity | 26.8% | 29% | 22.6% | 0.03 |
| Nadir CD4^+^ T-count, median (/µL) | 168 (41–295) | 175 (40–312) | 162 (47–265) | 0.14 |
| Current CD4^+^ T cells, median (/µL) | 439 (283–615) | 441 (284–607) | 439 (281–624) | 0.33 |
| ART – current use | 74.9% | 71.6% | 81% | 0.001 |
| HCV seropositivity | 26.8% | 29% | 22.6% | 0.03 |

AIDS, acquired immunodeficiency syndrome; ART, antiretroviral therapy; HCV, hepatitis C virus

**Online Resource 4.** Minor allele frequency of coding *EIF2AK3* SNVs

|  | Number of minor alleles | | |
| --- | --- | --- | --- |
| Allele | 0 | 1 | 2 |
| rs13045, A | 58.8% | 34.5% | 6.8% |
| rs867529, G | 69.1% | 26.1% | 4.8% |
| rs1805165, G | 69.1% | 26.1% | 4.8% |

SNV, single nucleotide variant

**Online Resource 5.** Concordance of coding *EIF2AK3* SNVs

|  | rs13045 | rs867529 | rs1805165 |
| --- | --- | --- | --- |
| rs13045 | - |  |  |
| rs867529 | 88.3% | - |  |
| rs1805165 | 88.3% | 100% | - |

SNV, single nucleotide variant

**Online Resource 6**. Distribution of *EIF2AK3* haplotypes in the targeted sequencing sub-cohort

|  | A/A | A/B | A/D | B/B | B/D | D/D |
| --- | --- | --- | --- | --- | --- | --- |
| No. participants (%) | 583 (58.7%) | 245 (24.7%) | 97 (9.8%) | 48 (4.8%) | 14 (1.4%) | 5 (0.5%) |
| Age, y (median) | 44 | 43 | 42 | 43 | 47 | 45 |
| Race |  |  |  |  |  |  |
| European | 33.2% | 64.5% | 38.1% | 75% | 92.9% | 20% |
| Hispanic | 6.9% | 14.7% | 8.3% | 20.8% | 0% | 60% |
| Black | 59.9% | 20.8% | 53.6% | 4.2% | 7.1% | 20% |
| Sex, female | 24.7% | 20% | 22.7% | 10.4% | 14.3% | 40% |
| AIDS diagnosis | 60.4% | 60% | 62.3% | 56.3% | 71.4% | 100% |
| Nadir CD4^+^ count, median (/mm^3^) | 164 | 189 | 165 | 200 | 76 | 171 |
| ART-experienced | 85.6% | 82.6% | 82.3% | 85.1% | 85.7% | 80% |
| HCV seropositivity | 28.4% | 22% | 24% | 17% | 35.7% | 40% |
| NCI, n (%) | 199 (34.1%) | 162 (66.1%) | 61 (62.9%) | 27 (56.3%) | 7 (50%) | 2 (40%) |

AIDS, acquired immunodeficiency syndrome; ART, antiretroviral therapy; HCV, hepatitis C virus; NCI, neurocognitive impairment; NP, neuropsychiatric

**Online Resource 7.** Association of *EIF2AK3* haplotypes with IL-6 concentrations in CSF **
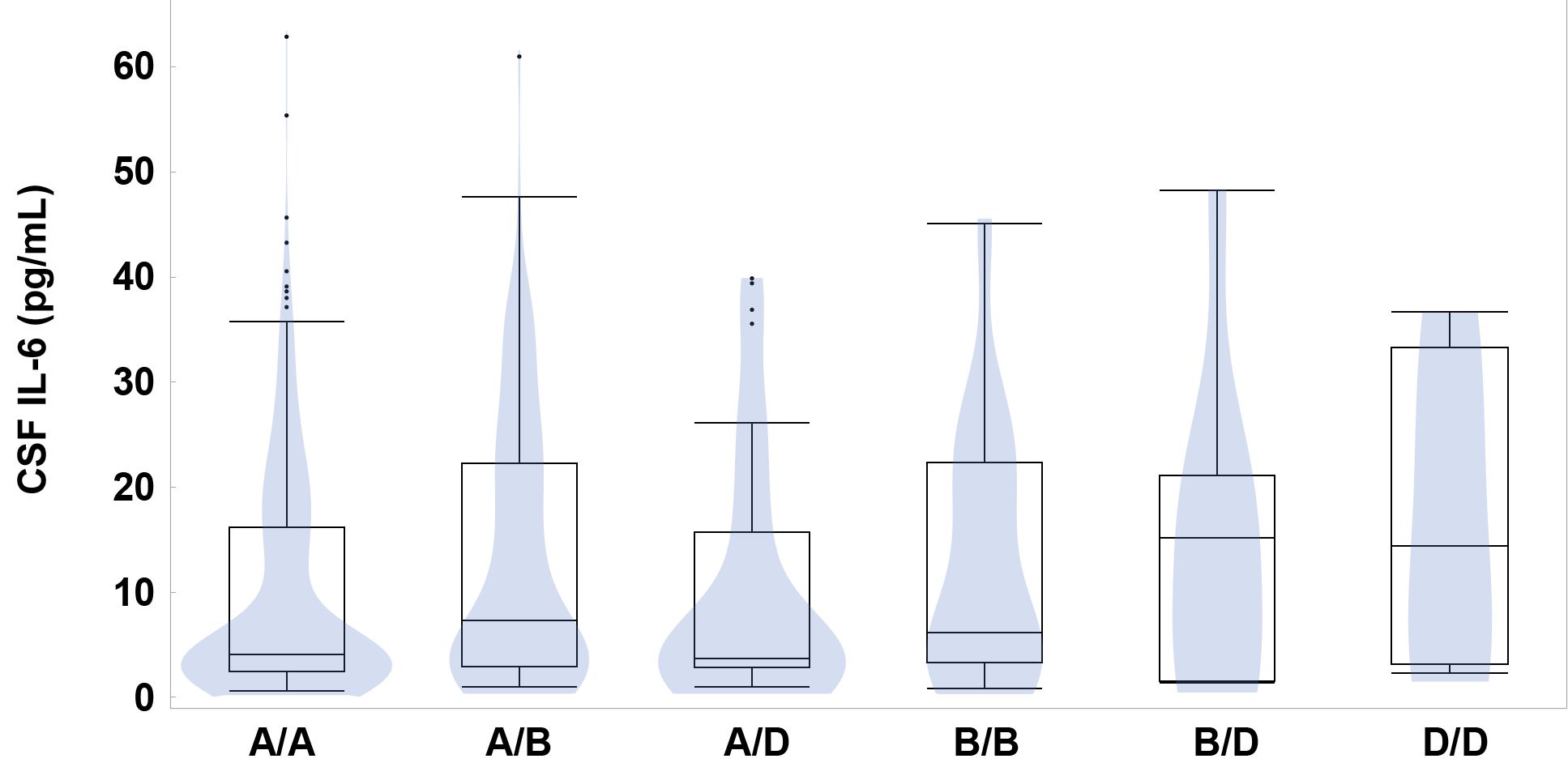
**

**Online Resource 8.** Global distribution of minor alleles of coding *EIF2AK3* SNVs

|  | Overall cohort | European | Asian | Latin | Black |
| --- | --- | --- | --- | --- | --- |
| rs13045 | 33% | 33% | 47%–51% | 28%–39% | 9%–15% |
| rs867529 | 26% | 26% | 39%–46% | 21%–31% | 1%–7% |
| rs1805165 | 27% | 27% | 41%–47% | 21%–41% | 1%–7% |

SNV, single nucleotide variant

**Online Resource 9.** Comparison of domain-specific deficit scores among groups categorized according to haplotypes

|  | Overall cohort | A/A | A/B | A/D | B/B | B/D | D/D |
| --- | --- | --- | --- | --- | --- | --- | --- |
| Verbal | 0.27 ± 0.02 | 0.24 ± 0.02 | 0.35 ± 0.04 | 0.2 ± 0.06 | 0.4 ± 0.08 | 0.21 ± 0.15 | 0.2 ± 0.25 |
| Executive | 0.6 ± 0.02 | 0.54 ± 0.03 | 0.66 ± 0.05 | 0.73 ± 0.08 | 0.99 ± 0.12 | 0.61 ± 0.22 | 0.8 ± 0.36 |
| Learning | 0.67 ± 0.03 | 0.67 ± 0.04 | 0.7 ± 0.06 | 0.67 ± 0.09 | 0.87 ± 0.13 | 1.07 ± 0.24 | 0.7 ± 0.4 |
| Recall | 0.52 ± 0.03 | 0.58 ± 0.04 | 0.55 ± 0.06 | 0.61 ± 0.9 | 0.5 ± 0.13 | 0.89 ± 0.23 | 1.1 ± 0.39 |
| Working memory | 0.48 ± 0.02 | 0.47 ± 0.03 | 0.38 ± 0.04 | 0.52 ± 0.07 | 0.6 ± 0.1 | 0.54 ± 0.19 | 0.7 ± 0.31 |
| Motor | 0.49 ± 0.03 | 0.38 ± 0.03 | 0.54 ± 0.05 | 0.41 ± 0.08 | 0.44 ± 0.12 | 0.69 ± 0.23 | 1 ± 0.37 |
